# Bayes Factors for Mixed Models: Perspective on Responses

**DOI:** 10.1007/s42113-022-00158-x

**Published:** 2023-02-14

**Authors:** Johnny van Doorn, Frederik Aust, Julia M. Haaf, Angelika M. Stefan, Eric-Jan Wagenmakers

**Affiliations:** Department of Psychological Methods, University of Amsterdam, Valckeniersstraat 59, 1018 XA Amsterdam, the Netherlands

**Keywords:** Bayes factors, Mixed effects, Mixed models, Random effects

## Abstract

In van Doorn et al. (2021), we outlined a series of open questions concerning Bayes factors for mixed effects model comparison, with an emphasis on the impact of aggregation, the effect of measurement error, the choice of prior distributions, and the detection of interactions. Seven expert commentaries (partially) addressed these initial questions. Surprisingly perhaps, the experts disagreed (often strongly) on what is best practice—a testament to the intricacy of conducting a mixed effect model comparison. Here, we provide our perspective on these comments and highlight topics that warrant further discussion. In general, we agree with many of the commentaries that in order to take full advantage of Bayesian mixed model comparison, it is important to be aware of the specific assumptions that underlie the to-be-compared models.

## Introduction

**Fig. 1:**
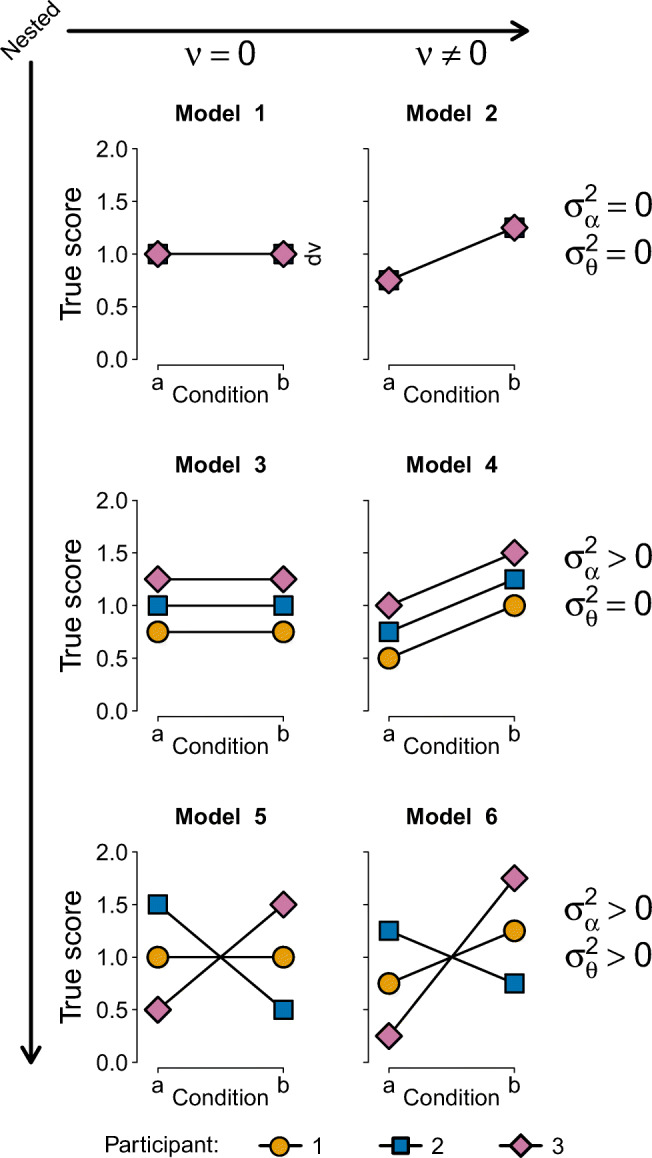
Prototypical patterns produced by six models, in the case where *I* = 3 participants each observe *M* = 1 trial in each of *J* = 2 conditions. Left column: models without a fixed effect of condition; right column: models with a fixed effect of condition. Top row: models without random effects; middle row: models with random intercepts only; bottom row: models with random intercepts and random slopes. The arrows indicate the nested structure: model complexity increases from left to right and from top to bottom. Figure from https://psyarxiv.com/y65h8 under a CC-BY license

In the target article on Bayesian mixed models, van Doorn et al. (2021) considered the prototypical scenario where participants respond to multiple trials across multiple conditions (e.g., control and experimental). In this scenario, several model comparisons can be made to determine whether there is a meaningful difference between conditions. Figure 1 illustrates six possible models that may be considered. We outlined a series of open questions concerning Bayes factors for mixed effects model comparison, with an emphasis on the impact of aggregation, the effect of measurement error, the choice of prior distributions, and the detection of interactions.

The target article was followed by seven commentaries (i.e., Rouder et al., 2021; Heathcote and Matzke, 2021; Singmann et al., 2021; Vasishth et al., 2021; Heck & Bockting, 2021; Linde & van Ravenzwaaij, 2021; Veríssimo, 2021). Many of these commentaries focused on one or several of the following three questions:

1. Which (if any) models ought to be excluded on a priori grounds?
2. How should we specify the prior distribution on effect size?
3. Is it possible to issue default recommendations when working with mixed effects models?

In broad strokes, it seems fair to say that all contributors agree that statistical practice (e.g., model specification and comparison) should be rooted in substantive considerations and tailored to answer the question at hand—mindless statistical rituals (Gigerenzer, 2004) must be avoided. Yet, there are some interesting points of disagreement about what this implies in practice. We believe that these disagreements reveal important considerations that the practical researcher should reflect upon when applying mixed models in their work.

In order to facilitate an open exchange of ideas, we initially refrained from addressing these questions ourselves. Instead, we reported simulation studies that highlighted the ramifications of particular modeling choices.

Below, we provide our perspective on the commentaries as they relate to the questions that were posed in the target article. The set of contributions to this topic will be concluded with a discussion article (cf. Shiffrin et al., 2021) that we hope will clarify outstanding disagreements.

## Response to the Seven Comments

Below, we first discuss Rouder et al. (2021), who support the *principle of marginality*; we then turn to Heathcote and Matzke (2021), who question the principle. This is followed by an extended discussion of Singmann et al. (2021), who offer both a specific critique of assigning prior distributions to standardized effect sizes and a general critique of Bayes factors. The Singmann et al. (2021) comment is echoed by Vasishth et al. (2021), who also argue against aggregation, against omitting the intercept-slope correlations, and in favor of a sensitivity analysis. Heck and Bockting (2021) propose to model-average across the competing models, such that models that predict the data relatively well have an increased influence on the resulting inference. Linde and van Ravenzwaaij (2021) present an extensive simulation study that further highlights the difference between the Strict null and the Balanced null comparisons. Finally, Veríssimo (2021) demonstrates that omitting random effects can result in Bayes factors that are overconfident.

### Rouder, Schnuerch, Haaf, and Morey

In a provocative article that focuses on the appropriate specification of models and prior distributions, Rouder et al. (2021) argue the following points:^1^

1. Common ANOVA procedures are fundamentally flawed.
2. It is important that models “faithfully translate theoretical constraints into formal or statistical constraints.”
3. Poorly specified models “exclude main effects but include their interactions. (Langsrud, 2003; Nelder, 1994; Venables, 2000; Yates, 1935)”
4. “the additive (i.e., Y~A+S) model should not be considered further.” The additive model refers to Model 4 in Fig. 1, that is, the model in which a true non-zero effect of condition is equally large for all participants. This implies that one of the key functions of the R package BayesFactor (i.e., anovaBF()) has effectively been deprecated.

Based on substantive considerations, Rouder et al. (2021) deem Models 4 and 5 (cf. Fig. 1) poorly specified and exclude them from consideration altogether. This stands in sharp contrast to both Heathcote and Matzke (2021), who argue against this exclusion, to Singmann et al. (2021), who take the inclusion of these models as self-evident, and to Heck and Bockting (2021), who model-average across the model ensemble.

Rouder et al. (2021) build logically on the premise that Models 4 and 5 are not theoretically meaningful. In the Rouder et al. scheme, the crucial comparison is therefore between Models 3 and 6, and this is affected by two elements: the fixed effect (i.e., the mean of the slopes) and the random effect (i.e., the variance of the slopes). Suppose we find strong evidence in favor of Model 6 versus Model 3: it is now unclear to what extent this evidence is caused by a fixed effect, a random effect, or both. Rouder et al. (2021) consider this ambiguity to be “more a feature than a limitation”.

More importantly, we believe that researchers are primarily concerned with the fixed effect, whereas the random effects are often just considered a nuisance. For instance, consider a paradigm in which there exists a small fixed effect that is dwarfed by large random effects (see their Scenario 1). When sparse data are obtained from this paradigm, we may only learn that there exist random effects. Model 6 may then decisively outperform Model 3, but this does not warrant the conclusion that there is good empirical evidence for the presence of a meaningful fixed effect—and this is exactly what we believe researchers wish to establish. It appears that Rouder et al. (2021) agree that strong evidence for a small fixed effect in the presence of a large random effect is uninteresting, as they “worry here if the mean is useful when a sizable proportion of individuals has a true effect in the opposite direction” (see also ; Haaf and Rouder, 2017). Yet, it is specifically in this case that the Strict null comparison yields substantially more evidence in favor of an effect than the Balanced null comparison (also see their Table 3). Despite the concerns of Rouder et al. about Model 5, the consequences of the Strict null comparison therefore seem to be more in line with their substantive expectations.

The a priori rejection of Models 4 and 5 also seems to demand that the modeler takes very seriously the existence of a point null hypothesis. In Model 4, the random effect is exactly zero, and in Model 5 the fixed effect is exactly zero. These radically excluded models appear to be back in contention when we assume instead that they are merely mathematically convenient representations of effects that in reality are very small. In this case, the choice of null model should by guided by the target on inference—individuals or population.^2^

To understand why, let us first consider why the ambiguous result of comparing Model 3 to Model 6 may be considered a feature. Model 6 assumes that the magnitudes of effects on each individual follow a normal distribution with some mean (the fixed effect) and variance (the random slope variance). In essence, Rouder et al. argue that there is little value in examining the mean of the distribution in isolation—researchers should examine the full distribution of effects. The reasoning is that the mean (expected value) of the distribution is relevant only to the degree that it is a fair summary of the population—when the mean is large relative to the variance. To understand why, consider a positive mean of effects of 0.5 with a standard deviation of 1. In this case, 30% of true individual effects are negative. If we relied on the mean to guess the direction of the effect in an individual, we would be wrong with a probability of 30%. So the benefit of the ambiguity^3^ in the strict null comparison is that it encourages the analyst to consider the full distribution of effects and to interpret the fixed effect estimate in relation to the variance of effects Haaf & Rouder (2017, 2019). Evidence in favor of Model 6 cannot be the end result; it simply indicates that something is happening that is worth investigating further.

Here, we get to the heart of the argument: If one is interested in inference at the level of individuals,^4^ it seems fair to say that it is of limited interest to test the fixed effect in the presence of random slopes (the balanced null). To claim an effect for the majority of individuals, it is most relevant whether the fixed effect, if present, is large relative to the random slope variance, not whether it differs from zero. Unfortunately, this question is not addressed directly by the strict null comparison. Rather the strict null comparison tests whether at least one individual shows an effect.

Yet, we think that a test of the fixed effect is relevant when one is interested in inference at the population level. Consider the following example. To address a lack of organ donors, it is prudent to study the effectiveness of switching from an opt-in to an opt-out approach: Will there be more organ donors if the default is that every adult donates? It is plausible that switching to opt-out will cause some individuals to become reactant and less willing to donate organs. Yet, the success of a change to public policy is largely determined by whether the new policy yields an overall increase in the number of donors. Conceivable opposite effects on a subset of individuals are of subordinate interest.^5^

To some degree, whether researchers are primarily interested in the individual- or population-level is an empirical question—one may simply survey a large group of researchers, explain Models 3–6, and query which comparisons they find most relevant.

We believe that Rouder et al. (2021) present compelling arguments for the exclusion of Models 4 and 5. However, we have some reservations. First, excluding these models, will be effectively prevented researchers from addressing the questions about population-level effects that they care about most. We speculate that researchers would be prone to misinterpreting answers in terms of the pertinent questions, that is, they would misinterpret compelling evidence for Model 6 over Model 3 as evidence for the presence of a fixed effect. Second, we worry that researchers interested in participant-level effects may stop at comparing Model 6 to Model 3 and fail to follow up evidence for Model 6 with a careful examination of the random effect distribution. We worry that researchers would not sufficiently consider whether “a sizable proportion of individuals has a true effect in the opposite direction”. For instance, consider a paradigm in which there exists a small fixed effect that is dwarfed by large random effects (see their Scenario 1). When sparse data are obtained from this paradigm, we may only learn that there exist random effects. Model 6 may then decisively outperform Model 3, but this does not warrant the conclusion that there is good empirical evidence for the presence of a meaningful fixed effect. Yet, it is specifically in this case that the strict null comparison yields substantially more evidence in favor of an effect than the balanced null comparison (also see their Table 3).

Relatedly, we worry that excluding Models 4 and 5 may enhance the fragility of the statistical conclusions under model misspecification. A single outlying participant suffices for Model 6 to outperform Model 3. A more sophisticated follow-up analysis (Haaf & Rouder, 2019, e.g.,) may then show that the result is due only to a single participant, but we worry that in practice such an analysis may often be forgotten or omitted, especially when they are not readily available.

Lastly, Rouder et al. (2021) provide important guiding principles on so-called fine-grained prior specification, showing some applications to the analysis of response times and Likert scale ratings. The recommendations made in this section are built on knowledge of comparable effects and standard deviations (e.g., the standard deviation being 200 ms in a typical priming experiment). The section concludes with the crucial reminder that the prior settings define the model being analyzed: there are no right and wrong prior specifications, only prior settings that create unrealistic models.

### Heathcote and Matzke

Heathcote and Matzke (2021) mainly focus on the question of whether certain models ought to be excluded a priori. In contrast to Rouder et al. (2021), Heathcote and Matzke (2021) argue strongly against the a priori exclusion of Model 5 (i.e., the model with only random effects and no fixed effect). They do so based on a different interpretation of some of the key literature on the marginality principle cited in van Doorn et al. (2021) and Rouder et al. (2021). Heathcote and Matzke (2021) argue that most of that literature does not apply to ANOVA models. For instance, Heathcote and Matzke (2021) state that “Venables (2000) draws a conclusion that one could certainly be forgiven in thinking generalizes to ANOVA models.” We are somewhat puzzled by the claim that the cited literature would not pertain to ANOVA, since the citation taken from Venables (2000) comes from a section that explicitly discusses factorial designs.

It seems that Heathcote and Matzke (2021) are focusing on the mathematical possibility of violating the principle of marginality (which, while perhaps slightly more difficult to compute, is trivial given today’s computational power), while the focus of the cited arguments in favor of marginality seem to be conceptual and theory-based. For instance, Nelder (1977) writes: “Thus, a S.S. [sum of squares] for A eliminating A.B [Model 5], though calculable given certain assumptions, makes no practical sense. I assert that the general rules for calculating S.S. must take account of marginality” (p. 50). This point is reiterated in other discussion papers as well (e.g., Kempthorne, 1975; Nelder and Lane, 1995; Herr, 1986), in addition to the more recent plea by Rouder et al. (2021). It is certainly possible to propose a research scenario where an interaction can be present without its constituent main effect. Proponents of the principle of marginality will argue, however, that these cases are exceedingly rare and therefore ill suited for default model comparison. Finally, we disagree with Heathcote and Matzke (2021) that the implausibility of Model 5 equals the implausibility of any other null-effect. For instance, the absence of a group difference strikes us as much more plausible than the presence of individual effects that just happen to balance out perfectly between two or more groups.

### Singmann et al.

In their commentary, Singmann et al. (2021) argue that researchers need to think deeply about the link between theory and the statistical models they implement. This connection between theory and models also includes the choice of prior distributions. While these two points are in perfect agreement with the line of argumentation by Rouder et al. (2021), Singmann et al. (2021) draw very different conclusions. Specifically, the authors advocate for priors on raw scales instead of standardized effect size, and are in favor of the Balanced null comparison.

#### General Remarks

Singmann et al. (2021) provide a central critique of the questions raised in the target article: They question whether it is useful at all to make default recommendations for statistical modeling. In their view, statistical modeling ought to be intricately tied to theoretical considerations and the “causal mechanisms” researchers aim to investigate. Therefore, the model specification, including prior distributions, cannot be separated from other components of empirical research. They summarize this observation as “Trying to make inferences from atheoretical modeling is, in effect, the tail (modeling) wagging the dog (theory)”.

We think that most of the researchers who contribute to the current debate on Bayesian linear mixed modeling would agree with this argumentation. For example, Rouder et al. (2021) note that “Models capture substantively relevant theoretical positions and allow for inferential statements”, and Heck and Bockting (2021) highlight that “researchers should be aware that auxiliary assumptions are required for translating substantive hypotheses to specific statistical models”. It therefore seems that the debate is not about whether model specification and model comparison should be based on theory, but to what degree statistical modeling can be similar across applications, which allows substantive researchers to use comparable modeling setups.

The question of whether any defaults are tenable, can be answered on many levels. An extreme version of the argument by Singmann et al. (2021) would be to abandon *t*-tests and ANOVA altogether, given that they represent default statistical methods. This extreme argument implies that any substantive research project would require a statistician on the team. Somewhat less extreme would be a proposal that statistical software for Bayesian methods should not have default prior settings, thus forcing researchers to make deliberate decisions. Regardless of whether or not this proposal is reasonable, it is certainly at odds with the reality of all current statistical software.

In general, Singmann et al. (2021) seem to be somewhat inconsistent in their consideration of when who wags what. While they argue against default model specification, they do argue in favor of Model 5 and the Balanced null comparison. In addition, many arguments in their commentary could be considered at best loosely connected to this line of argumentation. We will address their arguments from Part I and II of their commentary subsequently.

#### Remarks on Part I

In the first part of their commentary, Singmann et al. (2021) offer a thorough critique of the Bayes factor setup using JZS priors (Rouder et al., 2009; Rouder et al., 2012) for linear mixed models. This setup is currently used in many statistical software solutions including the R-package Bayes factor, JASP, and even SPSS. The key features of these default Bayes factors are (1) within each model, effects are standardized by the residual variance, (2) priors on residual variance and grand mean are uninformative Jeffreys priors, and (3) the JZS prior setup allows for a relatively easy and quick estimation of the Bayes factors, which have desirable statistical properties such as scale invariance and consistency. Yet, Singmann et al. (2021) highlight difficulties with interpretability of estimates and the choice of appropriate priors for linear mixed models.

##### The Issue of Priors and Aggregation

One question we posed in the target article aimed at how aggregation of data should be addressed. Singmann et al. (2021) tackle this question by proposing an alternative setup to the default Bayes factor, where effects are unstandardized.^6^ Aggregation affects the estimate of the residual variance. Therefore, if effects are standardized by the residual variance they, too, will be affected by aggregation. In contrast, unstandardized effects are unaffected by changes to the residual variance. Singmann et al. (2021) show that, if priors are not adjusted for aggregation, unstandardized models lead to unaffected Bayes factors while default Bayes factors are affected.

Singmann et al. (2021) argue appropriately adjusting priors for aggregation is difficult. When considering this argument, it is important to distinguish between two types of aggregation: complete and partial aggregation. Complete aggregation refers to the case where participants contribute only one observation to each level of the factor of interest. Partial aggregation, in contrast, reduces the number of observations but leaves at least two observation for each level of the factor. For illustration, consider the case of a 2×2 repeated-measures design: Following aggregation, each participant contributes one observation to each cell of the design. While this constitutes complete aggregation for the interaction term, multiple observation per participant remain for the analyses of the main effects—for the main effect of one factor observations are pooled over levels of the other factor. Partial and complete aggregation affect residual variance differently.

Partial aggregation reduces the error variance by a factor of ${1}/{\sqrt {n}}$. As also noted by Heck and Bockting (2021), the effect of aggregation on the resulting Bayes factors can be offset by adjusting the prior scales on fixed and random effects in the standardized parameterization by a factor of $\sqrt {n}$, i.e., $r\prime = r \sqrt {n}$. Aust et al. (2022) explore the effect of this adjustment for balanced null comparisons in a simulation. The results show that this prior adjustment works reasonably well. The only exception was in a simulation condition where an effect was present and the random slope variance was substantially smaller than the error variance. In this case, and when only two observations per cell remained, they observed an inflation of the Bayes factor. This inflation, however, was negligible for small and inconsequential for large Bayes factors. Thus, in most cases, the adjustment of priors for partial aggregation is simple and effective.

Adjusting priors for complete aggregation is more challenging. When data are fully aggregated, the random slopes variance collapses into the reduced error variance. In mixed models, the random slope variance is characterized by a probability distribution, which prohibits an exact adjustment of the prior by a simple scaling constant. Aust et al. (2022) explore the adequacy of an approximate adjustment using a point value for the random slope variance in a second simulation for balanced null comparisons. When the error variance is small or the number of participants is large, they find that the approximate adjustment works well. However, compared to the mixed model, aggregate analyses produced increasingly diverging Bayes factors when (1) the difference in random slope and error variance increased or when (2) the number of participants decreased. Thus, the adjustment of priors for complete aggregation is more difficult, especially with large error variances and few participants.

##### Interpretability Issues of Default Bayes Factors

Singmann et al. (2021) offer additional critique on the interpretability of standardized effects, and by extension, the default Bayes factor. In general, the residual variance in the linear mixed models considered here can be understood as within-participant trial-by-trial variability. Singmann et al. (2021) provide examples of more complex models—such as adding another nesting factor (crossed-random effects) or another trial-level covariate—where the interpretation of the residual variance, and therefore the specification of useful priors is much more difficult.

We agree that there are cases where the interpretation of effects that are standardized by the residual variance is difficult. However, estimates from models using the JZS prior setup can be easily transformed to unstandardized effects. In fact, this is done with all software solutions we know of. However, priors are still placed on the standardized effects, and it may be difficult to find reasonable scales for more complex models without much prior exploration. In these cases, it might be useful to use results from prior experiments, or to use prior predictive methods to make educated guesses.

Another matter discussed by Singmann et al. (2021) is how hierarchical shrinkage might affect standardized effects. In hierarchical models, the variance of condition effects is partitioned into an estimated random slope variance and residual variance due to noise. Therefore, the variance of the estimated random slopes is reduced as compared to the observed variance of condition effects if each person was modeled independently. In turn, estimated residual variance may contribute more to the total variance than in non-hierarchical modeling. Shrinkage therefore affects standardization of effect sizes estimates.

Here, it is important to be clear about what about what is meant by standardization: (1) standardization by the residual variance, as is used in the standardized model parameterization, and (2) standardization by the variance of condition effects, as is done in reporting study results (equivalent to Cohen’s *d*).^7^ If we standardize by the residual variance as is done with the standardized model parameterization, then effect size estimates are smaller than the observed equivalent. Note, however, that standardizing by residual variance is uncommon,^8^ so it seems unlikely that researchers will ever notice the effect of hierarchical shrinkage. If we standardize by the random slope variance as is typically done when reporting effect sizes, then effect size estimates are—sometimes considerably—larger than the observed equivalent. Whether effect size based on descriptive statistics are superior to the model-based equivalent, however, is up for debate. First, observed condition effect variances are contaminated by the standard error, i.e., the sample noise of the effect. Because the standard error depends on the number of trials, say, doubling the number of trials considerably increases effect sizes if trial variability and true random slope variability are kept constant. Therefore, model-based estimates are better comparable across studies with varying numbers of trials (Rouder and Haaf, 2019), and are more explicit as to which variance is used for standardization. We agree, however, that model-based effect size estimates do not conform to our intuitions about the size of standardized effects. These intuitions have mainly been shaped by Cohen’s dominant categorization into small, medium and large effect sizes (Cohen, 1988). Yet, these categories were never intended for within-subjects, massively nested designs, and it is therefore unclear whether our intuitions have any bearing for the current discussion.

##### Summary

Overall, some of the highlighted issues with the default Bayes factor may be addressed by adjusting prior scales on standardized effects. However, we agree that there are cases where the right degree of adjustment is difficult. In these cases, Singmann et al. (2021) recommend specifying unstandardized models. However, it may also be possible to adjust the JZS model specification to use a different error term for standardization. For example, we may use the total variance or some other combination of variance terms. Then, priors are placed on effect size units before partitioning the within-subject error variance. Exploring this option may be warranted in order to retain some of the key advantages of the default Bayes factors, namely that Bayes factors are well-behaved, invariant to changes in measurement scale, consistent, and computationally efficient (Rouder et al., 2012).

One question related to the critique by Singmann et al. (2021) is the general role of standardized effect sizes in estimation. This is a somewhat controversial issue. We agree that in an ideal world, researchers would use well-understood measures that are interpretable and meaningful as-is. In that case, it would be straightforward and preferable to use non-standardized estimates (either obtained from a model with standardized specification and transformed, or from an unstandardized model), but this is just not the case. We believe that most researchers will find it difficult to specify prior expectations about grand means, the magnitude of the error variance, and effect sizes, all on the raw scale.

#### Remarks on Part II

##### Mapping from Theoretical Constructs to Dependent Variables

The first topic of Part II concerns coordination, that is, the mapping function from theoretical constructs to dependent variables. We agree this is an important problem that has been unjustly ignored. Singmann et al. (2021) state that “a purely descriptive statistical framework predicated on the identification of main effects and interactions, such as the one laid out by vDAHSW, glosses over the problem of coordination (cf. Loftus, 1978; Wagenmakers et al., 2012)”. Indeed, results from standard statistical frameworks are tied to the measurement scale. As a solution, Singmann et al. (2021) propose to “place the understanding of theoretical constructs and their presumed coordinations at the forefront.”

We doubt that this recommendation is helpful for the practical researcher. Suppose that a mathematical psychologist carefully models a set lexical decision data with the drift diffusion model (Ratcliff, 1978). The results may show a non-crossover interaction of drift rates (e.g., in the standard task, older adults process lexical information more slowly than adolescents; when the letter strings are visually degraded, this slows down information processing more in older adults than it does in adolescents). The non-crossover character of the interaction means that its presence hinges on the extent to which the drift rate scale is of special interest. For instance, drift rates may stand in a non-linear relation to neural firing rates, and consequently the interaction may vanish when the data are expressed on the neural firing rate scale instead of on the drift rate scale. Evidently it was premature to conclude that visually degrading letter strings slows down information processing in lexical decision more in older adults than it does in adolescents. The problem does not end there, because neural firing rates themselves may non-linearly depend on, say, the chemical composition of the cells. Hence, expressing the data on this new scale may reintroduce the interaction. Maybe the original claim was correct after all. As argued in Wagenmakers et al. (2012, p. 156), “the problem is not to find a single proper scale—it may not exist—but to realize that conclusions that critically depend on the scale of measurement cannot be generalized to other scales.” Such a realization would be facilitated by using ordinal statistics, because these are invariant under monotonic transformations. As already pointed out by Jeffreys ((Jeffreys, 1961), p. 229), in psychology “there are few definite standards of measurement, but it is possible to arrange individuals in orders with respect to two or more abilities.”

##### Bayes Factors

The second topic of Part II concerns Bayes factors. Here, we take issue with several of the authors’ claims. First, they state that “As is well known, Bayes Factors work best when there is at least one of the considered models that well approximates the data generating process”. We agree in the sense that this claim applies to all statistical modeling—badly misspecified models may lead to unjustified conclusions (Anscombe, 1973; Matejka and Fitzmaurice, 2017). What the authors mean, however, is that Bayes factors are appropriate in the ${\mathscr{M}}$-closed setting (where one of the candidate models is true), and not in the ${\mathscr{M}}$-open setting. In our opinion, this idea is as popular as it is mistaken. The misconception is beguiling particularly because it provides a blanket excuse for disregarding the Bayes factor. Gronau & Wagenmakers ((Gronau & Wagenmakers, 2019), p. 37) have discussed this misconception, but we wish to reinforce their arguments here with two related observations:

1. The Bayes factor can be interpreted as the extent to which one model outpredicted the other for the observed data; specifically, the Bayes factor can be interpreted as a comparison of accumulative one-step-ahead prediction errors (Wagenmakers et al., 2006). As pointed out by Kass & Raftery (1995, p. 777): “The logarithm of the marginal probability of the data may also be viewed as a predictive score. This is of interest, because it leads to an interpretation of the Bayes factor that does not depend on viewing one of the models as “true”.”
2. The Bayes factor is a form of cross-validation (Fong & Holmes, 2020; Gneiting & Raftery, 2007). In fact, it is the only form of cross-validation that is coherent (i.e., does not result in contradictory conclusions). Cross-validation, of course, is a widely recommended technique to assess predictive performance in the ${\mathscr{M}}$-open setting (Browne, 2000).

Second, Singmann et al. (2021) refer to the work by Oelrich et al. (2020) and claim that the Bayes factor results in conclusions that are overconfident. We find this puzzling. Given the models and the data, the Bayes factor provides the unique measure of the strength of evidence. For instance, suppose we write computer code to generate binomial data, and we truthfully declare that the data have been generated either by ${\mathscr{M}}_{1}: \theta = 1$ or by a binomial chance parameter *𝜃* that is a random draw from a uniform distribution, ${\mathscr{M}}_{2}: \theta \sim U[0,1]$. The data are 5 successes and 0 failures. The corresponding Bayes factor is 6 in favor of ${\mathscr{M}}_{1}$ over ${\mathscr{M}}_{2}$. Presumably, there is widespread agreement that this Bayes factor is not overconfident, but rather the immediate and unique result of an exercise in probability theory. If this Bayes factor shows fundamentally different behavior than other methods of model comparison, this in fact dooms the other methods. The only escape from this conclusion would be to reject probability theory wholesale. We are not sure why the Bayes factor from the above simulation scenario would be broadly accepted as the only possible answer, whereas in real-life applications the Bayes factor would be judged to be “overconfident.”

In the Oelrich et al. (2020) manuscript, the overconfidence is assessed by considering data that could have been observed but were not—in the Bayesian world, such considerations are relevant only in the planning stage (e.g., Stefan et al., 2019); with the data in hand, inference ought to be conditional on those data. An analysis that does not condition on observed data is outside of the realm of rational belief updating.

Third, Singmann et al. (2021) argue that Bayes factors are difficult to apply in complex models. We agree, but we are unsure what concrete alternative the authors propose. Any statistical analysis can be improved, but this is a gradual process, and one that demands a consideration of the alternatives. And we firmly believe that the default Bayesian tests provide a worthwhile addition to the *p*-value reporting practice that still dominates our field.

#### Summary

In summary, Singmann et al. (2021) present a passionate plea to base statistical modeling on theoretical considerations, and highlight the relevance of good choices for implemented models and prior distributions. The authors point out difficulties with the parameterization of the models for the default Bayes factor, highlighting interpretation issues when effects are standardized relative to the residual variance. This is a valuable contribution to the debate around Bayesian linear mixed models.

Although we felt the need to push back on several points of critique, we are in general agreement with Singmann et al. (2021) when they conclude as follows:

“We believe that, with informed priors, Bayes factors can help adjudicate questions about the relative descriptive value of different models. Moreover, we believe that Bayesian methods are ideally suited to representing and propagating uncertainty in a principled way; this is especially useful in causal modeling, where joint posterior distributions over model parameters can be helpful in understanding the relationships between components of a potentially complex model and data. Rather, our target here is the often-tacit understanding of modeling as something that can be practiced fruitfully when distanced from the data and bound by constraints that are divorced from substantive theoretical considerations.”

### Vasishth et al.

Vasishth et al. (2021)’s article illustrates simulation-based design analyses for mixed models that can be used to determine the expected operational characteristics of a repeated measures design with a given sample size. Although experimental design was not a central concern in our target article, we agree that it is an important aspect of ensuring the informativeness of a study. Therefore, Vasishth et al. (2021)’s paper can be understood as a cautionary remark that a discussion about the informativeness of model comparisons should not be limited to the choice of models, but should include a more holistic perspective on the design.

Vasishth et al. (2021) also take a stance on some questions that we raised in the target article. In broad strokes, the authors urge not to rely on default assumptions but to specify tailored, appropriately complex hierarchical models that meaningfully represent theoretical assumptions about the subject under study. From this position, the authors argue that (1) random effects for items should be modelled and, therefore, data should never be aggregated, (2) correlations between random intercepts and slopes should be modelled, and (3) unstandardized effect sizes are, with few exceptions, preferable to standardized effect sizes. Thus, their position is well in line with Singmann et al. (2021), who similarly suggest that relying on defaults and standardizing effect sizes is undesirable. We have commented on these issues above, but in the following we want to add some comments prompted by Vasishth et al. (2021).

As previously noted, we agree that statistical models should be tailored to a specific research context. However, in our view, there are at least two instances in which default priors can be useful. First, in practice it may be difficult to specify appropriately informed priors. In this sense, the exemplary analysis presented by Vasishth et al. (2021) depicts a best-case scenario. The authors analyze data from a psycholinguistic experiment on the difference in processing of subject and object relative clauses, “perhaps the single most studied syntactic construction in psycholinguistics” (p. 4). The authors argue that it is important to use substantively informed prior distributions and accordingly carefully craft a prior for the mean difference of primary interest and for the intercept. In doing so, the authors draw on the results of a meta-analysis on the topic. Clearly, specification of informed priors is relatively easy for such intensely studied topics. However, in our opinion, when venturing into less charted territory (new materials, paradigms, and research questions), the usefulness of defaults cannot be entirely dismissed. Second, default priors can also be useful for parameters that are not of primary interest. Consider the priors (Vasishth et al., 2021) chose for random effect variances and correlations. In contrast to intercept and mean difference, these priors appear to be less informed and more default-like: Participant and item random intercept and slope variances all receive identical priors that had originally been specified in a different context (Schad et al., 2021). Similarly, the prior on the random effects correlations is chosen to be “diffuse” (i.e. an LKJ distribution with 2 degrees of freedom Schad et al., 2021, p. 113) with no theoretical considerations.

Vasishth et al. (2021) take a strong stance on aggregating data: It “should in general *never* be done” (p. 2). The key argument is that aggregating data ignores random item effects and thereby prohibits generalizing results to the population of items. We agree that where a sufficiently diverse sample of items is used, random item effects should be modelled just as any other important source of variance. That said, questions about aggregation remain relevant. First, in some paradigms random item effects may not be applicable: Researchers may assign each participant a different set of (e.g., randomly generated) items such that item effects are absorbed by the residual error variance. For other paradigms, the notion of sampling at random from a (normally distributed) population of stimuli may not be applicable, for example, the random dot motion task or flanker and Simon effects. Second, even when including crossed random effects for participants and items it may still be possible to aggregate. If participants respond repeatedly to each item (e.g., Stroop task), trial-level data can be aggregated for each participant-item combination before modelling random effects for both participants and items. Indeed, there may be good reasons to aggregate observations: When it is not clear that the dependent variable is well described by a parametric distribution, aggregation can serve as a safeguard against misspecification when the averaged observations can be assumed to be approximately normally distributed according to central limit theorem. Whenever data are aggregated, it is, however, important to be aware of potential biases that can arise (Schad et al., 2022).

On the topic of intercept-slope correlations, we agree that these can be theoretically interesting in many research contexts. If so, these correlations clearly should be modelled. Vasishth et al. (2021) critique that all models specified in the target article assume random effects to be uncorrelated—this assumption is made for all models available in the *BayesFactor* package. Is this assumption problematic when fixed effects are of primary interest—as was the case in our target article? This depends on which model comparison is performed. Relying on models that omit intercept-slope correlations does not affect the Balanced null comparison. Random slopes and the intercept-slope correlation are assumed in both the null and alternative model. Hence, the magnitude of the correlation does not affect the inference (Barr et al., 2013; Linde and van Ravenzwaaij, 2021). Adding intercept-slope correlations will, however, affect the Strict null comparison.^9^ This is because the null model, in contrast to the alternative, assumes no random slope variance and consequently no intercept-slope-correlation. This Strict null comparison therefore tests three terms simultaneously: (1) the mean difference, (2) participant random slopes, and (3) the correlation between participants’ random intercepts and slopes. Thus, if the data are generated with random slope variance and intercept-slope correlation, the evidence for a condition difference should increase with the magnitude of the correlation. We thank Vasishth et al. (2021) for motivating the addition of random effect correlations to the model specification. We agree that a modeling approach that allows for intercept-slope correlations might be preferable and more broadly applicable. With regard to the question of which model comparison is most appropriate, we suspect that adding the intercept-slope correlation does not change the arguments brought forth by proponents of the Strict and Balanced null comparisons.

Finally, in line with Rouder et al. (2021), Vasishth et al. (2021) emphasize the importance of Bayes factor sensitivity analyses. We endorse this suggestion, and believe that it is particularly useful if non-standard modeling decisions are taken. However, we also believe that it raises the question of which modeling decisions should be subjected to a sensitivity analysis. Vasishth et al. (2021) seem to apply the concept of sensitivity analyses only to prior distributions, but theoretically other targets could be chosen as well, such as sampling priors in design analyses, effect size representations, or likelihoods. There are few guidelines for sensitivity analyses in Bayesian hypothesis testing so far (e.g., Sinharay and Stern, 2002; Stefan et al. in press; Schad et al., 2021; Schad et al., 2022), and we believe that it could be fruitful to devote more attention to the topic.

### Heck and Bockting

Heck and Bockting (2021) make a case for Bayesian model averaging, where all pertinent models are applied to the data simultaneously, and the relative impact on the resulting inference is determined by each model’s posterior probability. This also allows an empirical evaluation of the theoretical claim that particular models should be excluded from consideration altogether.

Model averaging as applied by Heck & Bockting does not circumvent considerations about which models are “well-specified” (Rouder et al., 2021) and which models to compare.^10^ As one possibility, Heck & Bockting propose a model averaged comparison to test for a fixed effect that pits Models 3 and 5 (neither contain the fixed effect) against Model 4 and 6 (both contain the fixed effect). With infinite data, there are two possibilities, depending on the presence of random slopes. If random slopes are present, the model averaged comparison will be dominated by Models 5 and 6, and thus becomes the Balanced null comparison (violating the principle of marginality). If random slopes are absent, the comparison will be dominated by Models 3 and 4, and thus becomes the RM ANOVA comparison (violating the maximal principle, although this principle could be irrelevant in such a case). Hence, the comparison proposed by Heck & Bockting sides with the Balanced null comparison. It is, however, straightforward to set up an alternative model averaged comparison to test for a fixed effect that respects the principle of marginality: If Model 5 is excluded a priori, with infinite data the comparison will become either the Strict null comparison, or the RM ANOVA comparison. It seems then that model averaging alone does not resolve the issue of which models to compare.

Additionally, we note that this point extends beyond the debate about the appropriate null model. In their article, Heck & Bockting also make less controversial exclusions. For instance, the model with only random slopes, and no random/fixed intercepts and fixed effect, is excluded. While most will agree that this is a nonsensical model, it highlights the large number of mixed effects models that are possible, even with only one predictor variable. Each of these models still needs to be assigned a prior probability in Bayesian inference (which may be zero, thus excluding a model a priori), irrespective whether focusing on model selection or model averaging.

### Linde and van Ravenzwaaij

Linde and van Ravenzwaaij (2021) expand on the findings of Case 1 in van Doorn et al. (2021), where the three different comparisons are demonstrated to lead to different conclusions, and are affected differently by the process of aggregation. In their article, Linde and van Ravenzwaaij (2021) revisit this scenario with an extensive simulation study, where various parameters (random intercept variance, random slope variance, fixed effect magnitude, and correlation between random intercepts and random slopes), sample characteristics (number of items, trials, and participants), and prior specifications (prior width for fixed effects and for random effects) are explored. The simulation results underscore the key difference between the Strict null and Balanced null comparisons: when increasing the random slope variance and/or the number of trials per participant, the Strict null quickly grows more diagnostic (or, overzealous), since only one of its models contains the random slopes component.

Interestingly, there appeared to be no differences in how the comparisons were affected by adjusting the prior width for the random effects components. We would expect the Strict null comparisons to be affected more extremely than the other comparisons, although this might be attributed to the simulation study varying the prior scale for both the random slopes and random intercepts simultaneously.

Linde and van Ravenzwaaij (2021) conclude with a call-back to Jeffreys’ platitude:^11^ “Bayes factors are not magic. They can answer the question researchers ask. In that sense none of the three comparisons is strictly superior to any other.” We believe that few researchers would want to argue against this notion.

### Veríssimo

Instead of investigating the choice of null model, Veríssimo (2021) provides more background on the importance of employing the maximal model as the alternative model when there are multiple predictor variables of interest. While most of the articles discussed so far focus on whether to include random slopes but no fixed effect for a predictor variable (i.e., the tenability of Model 5), Veríssimo (2021) discusses the consequences of not adding the corresponding random slopes to a fixed effect. Specifically, Veríssimo outlines several possible model comparisons in the scenario where two predictor variables are included in the model. When there is a correlation between the two predictor variables, and random slopes are only included for one of the variables, there will be a *mismatch* between the fixed and random components in the model. Such a mismatch will lead to overconfident Bayes factors when the true model includes random slopes for both predictor variables. The model comparison used to illustrate this behavior is the Balanced null comparison. While the conclusions of Veríssimo (2021) offer no arguments for choosing the Balanced null comparison over the Strict null comparison, the commentary highlights the recommendation to keep it maximal.

## Discussion

What is best practice for analyzing mixed models with Bayes factors? The discussion on van Doorn et al. (2021) revealed interesting disagreement among experts. This is not necessarily something to lament, as statistical choices are to some extent guided by philosophical preference and theoretical considerations rather than mathematical facts. Additional experience with Bayes factors for mixed models may be needed before more consensus is reached. All contributors would probably agree that, regardless of what method is used for inference, it should be tailored to the question at hand. The commentaries also confirmed that trying to entice researchers to address the same questions is like herding cats: the discussants generally pursued their own interests.

While only one submission actively endorsed the a priori exclusion of Model 5, there were also not many active opponents of the exclusion. Most submissions appeared to passively accept the tenability of Model 5. The common ground (at the cost of its extreme broadness) among the submissions is that each model (comparison) has different theoretical implications, and that the researcher ought to be aware of each. Not even model averaging can replace the requirement of thinking.

Similarly, specifying the prior distribution on the standardized effect has been demonstrated to be a dangerous pitfall. Using the raw effect size might be more advisable here, since it explicitly makes the analyst think about the appropriate scale of the prior distribution, rather than mindlessly accepting a default value. Alternatively, one could keep the standardized specification but remove the default scale settings on effects from statistical software, encouraging substantive researchers to at least make an active commitment.

With these two ideals in mind, however, we arrive at reality. While everyone will agree that both statistical and theoretical expertise will make for the most robust inference, there might be cases where one or the other is lacking. The researched phenomenon might be too novel to yield previous results to base a prior distribution on, or there might be no resources to hire a seasoned statistician to create a tailor-made statistical model. Or the seasoned statistician might be interested in developing a statistical software or R-package, where default values are built-in. In these cases, we would argue that some starting principles are in order, in terms of a default prior scale, or model comparison procedure. At the very least, however, we can write papers that explain what the default values mean. We can explore what a specific model comparison implies, in a general way, such that the mixed modeler can make a more informed choice in their own specific scenario.

We hope that this debate has brought a number of critical assumptions in mixed modeling to the foreground. Awareness of these assumptions is a necessary first step toward employing the methodology that best matches a particular research question and a particular philosophical preference.

## Data Availability

Not applicable.

## Footnotes

1 This response to Rouder et al. (2021) has been written without the input of Julia Haaf.

2 Note that because we worked in parallel on portions of this manuscript and the forthcoming discussion article in the current issue, the arguments made here are reiterated in the discussion paper.

3 This ambiguity is not, in principle, irreducible in the Rouder et al. scheme. If Model 6 is favored over Model 3, the contribution of random slope variance could be tested conditional on the presence of a fixed effect by comparing Model 6 to Model 4 without violating the principle of marginality.

4 Rouder et al. direct their argument at cognitive psychologists (p. 8), who typically aim at inferences relevant to the large majority of individuals.

5 In medicine, an analogous distinction is made between two targets of inference: conditional and marginal treatment effects (e.g., Remiro-Azócar et al., 2021). It is accepted that each has their place. Conditional treatment effects are relevant in clinical practice when a decision is made for a given patient (conditional on their covariate manifestations), whereas marginal treatment effects are relevant to public policy, which affects the population as a whole.

6 As pointed out by Singmann et al. (2021), the formulas for each of the models in the original article by van Doorn et al. (2021) are presented using the unstandardized effect size parameterization. While these models are mathematically equivalent up to the prior distributions (Haaf and Rouder, 2017, p.783), the standardized parameterization may highlight better how aggregation affects model comparison. We refer readers to Haaf & Rouder (2017, p.783) for a more detailed discussion about the standardized parameterization.

7 We note that the issue of standardized versus unstandardized effect size is discussed more extensively in the forthcoming discussion article in the current special issue.

8 To compute the “observed” residual variance, one would need to compute the within-person variance for each person and then pool these variances. We are not aware that this is a common procedure.

9 Random effect correlations cannot be meaningfully modelled in the RM-ANOVA comparison.

10 Model averaging is also not limited to Bayes factor hypothesis testing: it can also be valuable for parameter estimation (van den Bergh et al., 2021).

11 “The most beneficial result that I can hope for as a consequence of this work is that more attention will be paid to the precise statement of the alternatives involved in the questions asked. It is sometimes considered a paradox that the answer depends not only on the observations but on the question; it should be a platitude.” (p. vi ; Jeffreys, 1939)
